# Knowledge and Behavioral Practice of Mothers About Childhood Diarrhea in Arar City, Saudi Arabia

**DOI:** 10.7759/cureus.54221

**Published:** 2024-02-14

**Authors:** Hanaa E Bayomy, Hanan M Almatrafi, Sarah F Alenazi, Rehab Madallah S. Almatrafi, Miad Alenezi, Waleed A Alanazi

**Affiliations:** 1 Family and Community Medicine, Northern Border University, Arar, SAU; 2 Public Health and Community Medicine, Faculty of Medicine, Benha University, Benha, EGY; 3 College of Medicine, Northern Border University, Arar, SAU; 4 College of Medicine, Northern border University, Arar, SAU; 5 Pediatric Medicine, Maternity and Children Hospital, Ministry of Health, Arar, SAU

**Keywords:** arar city, children under five, behavioral practices, diarrhea, mothers, knowledge

## Abstract

Background: Diarrhea is the second most significant cause of child morbidity and mortality, especially in developing countries. The World Health Organization (WHO) advises that mothers and other caregivers be able to recognize the symptoms of dehydration. Therefore, this study aimed to assess the knowledge and behavioral practices regarding diarrhea among mothers in Arar City, Saudi Arabia.

Methods: This cross-sectional survey used an anonymous online questionnaire distributed among mothers of children aged one to five years in Arar City. The snowball convenient sampling method was used to recruit the participants. Information on knowledge and behavioral practices regarding diarrhea was obtained from the mothers of children through an electronic questionnaire. The Chi-square test and Fisher's exact test were used to evaluate the relationship between studied variables, as appropriate with statistical significance at P<0.05.

Results: A total of 479 mothers participated in this survey. Of these, 421 were included in the analysis. Most mothers fall within the age range of 20-40 years (71.1%). A large sector of the studied mothers had high education (72.4%) and was a housewife (40.4%). Most children were above one year old (77.7%). Most participants (69.6%) fell into the moderate knowledge category and 56.3% had moderate behavioral practice scores. Maternal education was significantly associated with knowledge. Furthermore, maternal education and behavioral practice levels were significantly correlated (P < 0.01).

Conclusion: The findings highlight the importance of targeted education programs and community-based interventions to improve mothers' knowledge and promote appropriate behavioral practices related to childhood diarrhea that ultimately will lead to improved health outcomes for children globally.

## Introduction

Diarrheal disease is characterized by frequent, loose, or watery stools [1]. Various factors, including infections, viruses, bacteria, parasites, certain medications, food intolerances, and underlying medical conditions can cause it [2,3]. Diarrhea is classified as either acute (loose stools <14 days) or chronic (diarrhea lasting>14 days and symptoms lasting >30 days) [4]. It is one of the widespread diseases affecting infants and children's growth and development [5]. Diarrhea remains a significant global health issue, particularly in developing countries with limited access to clean water, sanitation facilities, and proper healthcare [6-8]. It is considered the world's second-leading cause of mortality and morbidity in children [6]. Every year, over two million children under five die from diarrhea globally; 80% of this mortality occurs during the first two years of life [9]. According to the World Health Organization (WHO) and UNICEF, diarrhea affects 2 billion people worldwide each year, and 1.9 million children under the age of five die from diarrhea yearly, mainly in developing countries [10,11]. Diarrhea kills more children globally than AIDS, malaria, and measles combined [12,13]. While diarrhea-related childhood mortality rates have decreased in recent years, it remains a leading cause of death among children under the age of five [11]. Efforts to improve access to clean water, promote proper sanitation and hygiene practices, encourage breastfeeding, and provide oral rehydration therapy (ORT) have contributed to the reduction in mortality rates [14,15].

Changes in bowel motions diminish the consistency of fecal output, resulting in feces with more water in the stools than typical [16]. Diarrhea causes symptoms such as vomiting and diarrhea and can quickly lead to dehydration in young children [17]. Dehydration symptoms include thirst, irritability, restlessness, lethargy, sunken eyes, dry lips and tongue, dry skin, and fewer trips to the bathroom to pee [18]. In severe cases, it can lead to electrolyte imbalances and weight loss [19].

Diarrhea can be caused by a range of factors, including gastrointestinal infections (such as rotavirus, norovirus, or *Escherichia coli*), contaminated food or water, poor sanitation and hygiene practices, malnutrition, weak immune systems, and certain underlying medical conditions (such as inflammatory bowel disease or irritable bowel syndrome) [20,21]. These factors are lacking in Saudi Arabia due to the hot and dry climate, which is averse to diarrheal diseases [22]. Furthermore, the population can access clean drinking water and hygienic latrines [23]. According to a study conducted in Saudi Arabia, environmental risk factors for diarrhea in children included eating out after school hours, using reusable towels or sponges to dry dishes, and sewage leaking close to the house [24,25]. However, the estimated incidence rate of diarrhea in children under two years in Saudi Arabia is 25%, which is comparable to the global norm [26].

Efforts to combat diarrhea have yielded positive outcomes. There has been a notable reduction in childhood mortality rates, attributed to interventions such as improved access to clean water, sanitation facilities, and ORT [27,28]. Vaccination programs, especially the rotavirus vaccine, have shown promising results in reducing severe diarrhea cases [29]. However, challenges persist in resource-limited settings, necessitating the development of sustainable interventions tailored to local contexts. Also, harmful activities by mothers, including limiting nutrition, avoiding breastfeeding, and using unsuitable conventional therapy or incorrect prescriptions, have been reported [25]. Improving mothers' understanding and demonstrating good practice is essential for avoiding or preventing the spread of diarrhea [24,25]. One of the essential initial measures to prevent diarrhea's possible effects, such as dehydration, is managing acute diarrheal illness at home [30]. The prevention of diarrhea-related problems is significantly influenced by the parents' knowledge and behavior toward managing diarrheal diseases [31]. Therefore, this study aimed to shed light on the knowledge and behavioral practices regarding diarrhea among mothers in Arar City, Saudi Arabia. By identifying the level of awareness, health promotion, and prevention programs would be arranged to meet these knowledge gaps.

## Materials and methods

Study setting

In Arar City, Saudi Arabia, mothers of children under five years old participated in a cross-sectional survey. Data collection was collected between August and October 2023.

Study opulation and sampling methods

The participants were recruited using snowball sampling approaches and convenience. The study's eligibility requirements included being a resident of Arar City, possessing a computer or mobile phone, being educated enough to fill out the survey on their own, and having children under the age of five.

Sample size

The minimal sample size was calculated using the following formula [32]:

Sample size= (Z_1-__α/2_)^2 ^P(1-P)/d^2^

Z_1-α/2 _= is the standard normal variate at 5% type 1 error (P<0.05); it is 1.96,

P = the expected proportion based on previous studies,

d = the absolute error (0.05).

The expected proportion of was considered 50% since there are no previous studies in the Northern Border region, and to maximize the sample size. Allowing for a 10% non-response rate, the calculated sample size was 422 (384+ (384x10%)).

Tools of data collection

Data were gathered using a standardized questionnaire using Google Forms and distributed by email and several social media platforms, such as Facebook and Twitter (WhatsApp), developed after a thorough examination of the literature [33,25]. The questionnaire is divided into three sections. The first section gathers information about health and sociodemographic factors, such as age, education, occupation, gender, and age of the children. The second section collected knowledge data about the definition of childhood diarrhea and its common causes, including viral and bacterial infections, contaminated water and poor sanitation, sensitivity to some foods, and medications (13 items). Furthermore, it collected general knowledge about the clinical picture of diarrhea (seven items), preventive measures such as proper handwashing, exclusive breastfeeding, ensuring a clean water supply, and vaccinations (six items), and the treatment of diarrhea, including antibiotic use, oral rehydration solutions, continued breastfeeding, herbs, solid food, rest, and constipating drugs (12 items). The third section collected data about the behavioral practices of diarrhea among Arar mothers (15 questions; i.e., using antibiotics or oral rehydration salts (ORS) while treating diarrhea, giving ORS once a day or several times a day, dissolving ORS in juice or clean, sterile water, treating diarrhea with herbal preparations or bed rest, treating diarrhea in the hospital or at home, using solid foods or constipating medications to treat diarrhea, stopping breastfeeding during diarrhea, washing hands, vegetables, and fruits, and sources to obtain information about children's diarrhea?).

Each correct answer in the given questions received a score of 1, while an incorrect answer was assigned a score of 0. The total score was then multiplied by a factor to achieve a standardized scale ranging from 0 to 100. This adjustment ensures that the maximum achievable score is 100, while the minimum is 0 (see Appendices).

Plan of data collection

A team of researchers used messaging and social media sites to disseminate an anonymous online poll. An electronic version of the survey was made. Before beginning data collection, the research team conducted a pilot study to verify the web tool's viability and accessibility. They asked each collaborator to provide a minimum of two responses.

Ethical considerations and approval

The study was approved by the Local Committee of Bioethics (HAP-09-A-043), College of Medicine, Northern Border University, Saudi Arabia (IRB number: 56/44/H). The study was performed by the ethical standards laid down in the 1964 Declaration of Helsinki and its later amendments or comparable ethical standards. Before the survey was administered, each participant received information on its voluntary nature and was asked to provide consent by answering a question on the survey front page. Every participant was given the option to "agree" or "not to agree" to take part in the study. The responses were stored in a password-protected computer that was only accessed by the lead investigator, ensuring data confidentiality.

Statistical analysis

Statistical Package for the Social Sciences (IBM SPSS Statistics for Windows, IBM Corp., Version 17.0, Armonk, NY) was used to handle and analyze the data. Whereas nominal and categorical variables were described by frequency and percentage, numerical variables were described by the mean and standard deviation (SD). To evaluate the relationship between the categorical variables, the Chi-square test and Fisher's exact test were employed, as appropriate. Sensitivity analysis was performed by identifying the independent variables among knowledgeable individuals. The overall knowledge was categorized, using Bloom’s cut-off point, as good if the score was between 80% and 100%, moderate if the score was between 60% and 79%, and poor if the score was less than 60%. A p-value < 0.05 was considered statistically significant.

## Results

A total of 479 mothers participated in this survey, and 421 of them were included in the analysis. The remaining were excluded due to different causes including incomplete or inconsistent data or having children aged above five years. Their mean age was 33.4±8.7 years. More than two-thirds were aged between 20 and 40 years (Table 1). More than half of their children were males (57.5%) and above two years (51.3%). Most studied mothers had higher education (72.4%) and only 2.9% did not have educational certificates. The highest proportion of mothers were housewives (40.4%) followed by clerks (38.9%).

**Table 1:** Sociodemographic criteria of studied mothers

Studied variables (n = 421)		Frequency	Percent
Age of mother	< 20 years	14	3.3
	20 years	136	32.3
	30 years	155	36.8
	≥ 40 years	116	27.6
	Mean± SD	33.4±8.7	
Child age	less 1 year	96	22.8
	1-2 year	108	25.7
	above 2 years	216	51.3
Education	No educational certificate	12	2.9
	Secondary or below	104	24.7
	Bachelor's degree and postgraduate	305	72.4
Occupation	Clerk	164	38.9
	Healthcare worker	26	6.2
	Housewife	170	40.4
	Student	61	14.5
Children sex	Female	179	42.5
	Male	242	57.5

Table 2 provides descriptive statistics for the knowledge and behavioral practices of the studied mothers. The mean score of the standardized overall knowledge score was 64.3 ± 9.6 ranging from 34.2 to 89.5. Knowledge regarding the prevention of diarrhea, the score ranges from 0.00 to 6.00, with a mean of 4.8 and a standard deviation of 1.6. The knowledge of treatment of the diarrhea domain ranged from 3.00 to 12.00, with a mean of 8.3 and a standard deviation of 1.6. The score of knowledge about clinical pictures ranged from 0.00 to 6.00, with a mean of 3.1 and a standard deviation of 1.5. The score of the definition of diarrhea and mode of transmission ranges from 3.00 to 12.00, with a mean of 8.2 and a standard deviation of 1.6. The score of attitude and behavior ranged from 33.0 to 100.00, with a mean of 68.6 and a standard deviation of 12.3.

**Table 2:** Description of knowledge and behavioral practices of studied mothers

Descriptive statistics		Minimum	Maximum	Mean	SD
Knowledge	The overall score	34.2	89.5	64.3	9.6
	Prevention	0.0	6.0	4.8	1.6
	Treatment	3.0	12.0	8.3	1.6
	Clinical picture	0.0	6.0	3.1	1.5
	Definition and mode of transmission	3.0	12.0	8.2	1.6
Behavior and practice		33.0	100.0	68.6	12.3

**Figure 1:**
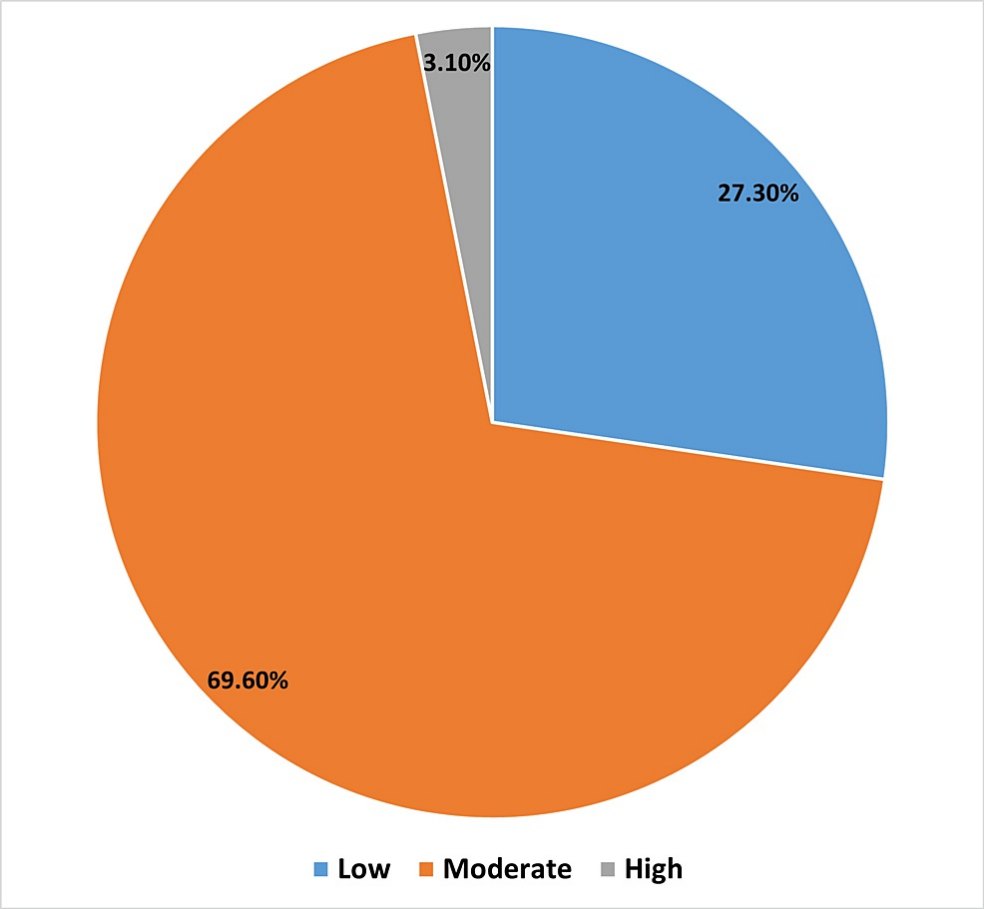
Distribution of the studied participants based on their knowledge score

Figure 1 illustrates the distribution of the studied participants based on their knowledge scores. A notable percentage of participants fell into the low knowledge score category of (27.3%, n=115). Most participants (69.6%, n=293) fall into the moderate category. A very small percentage of participants (3.0%, n=13) fall into the high knowledge score category.

Most mothers reported that their main source of information about health issues was healthcare workers (HCWs) (65.8%, n=277), followed by friends and relatives (40.9%, n=172). A minority got information from the internet (18.3%, n=77), television (15.2%, n=64), and books (10.9%, n=46).

**Figure 2:**
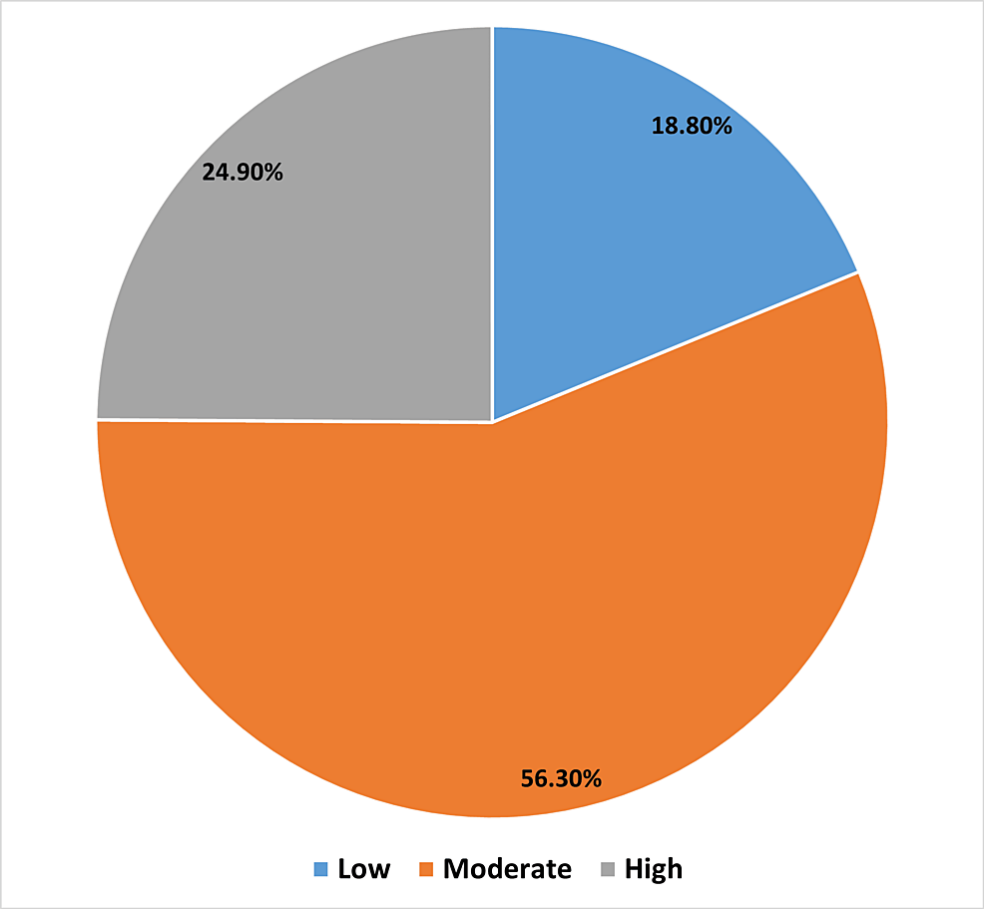
Distribution of the studied participants based on their behavioral practice score

Figure 2 presents the distribution of the studied participants based on their practice scores. A relatively small percentage of participants have low attitude scores (18.8%, n=79). The majority of participants (56.3%, n=237) fall into the moderate category. A significant but smaller percentage of participants (24.9%, n=105) have high attitude scores.

Table 3 compares knowledge levels (low, moderate, high) based on different demographic variables (age, education, and occupation) among the studied participants. Participants under 20 years had the lowest proportion of high knowledge (0.00%), while mothers aged 30-40 years had the highest proportion of high knowledge (4.5%, n=7). However, this difference was not statistically significant (p = 0.728). Participants with no educational certificate show a significantly higher proportion of low knowledge (66.7%, n=8) compared to moderate (33.3%, n=4), and high (0.00%). Other education groups (secondary or below, and bachelor’s degree and postgraduate) have varying distributions. This difference was statistically significant, p = 0.048. HCWs show a higher proportion of high knowledge (11.5%, n=3) compared to other occupation categories, nonetheless, this difference was not statistically significant (p = 0.233). The source of information did not differ significantly across the knowledge of the studied mothers except for HCWs (p<0.001) and the TV (p = 0.037).

**Table 3:** Association between socio-demographic characteristics and level of knowledge A p-value<0.05 was considered statistically significant.

Sociodemographic factors (n=421)		Low		Moderate		High		p
		No.	%	No.	%	No.	%	
Age	< 20 years	6	42.9	8	57.1	0	0.00	0.728
	20 years	34	25.0	99	72.8	3	2.2	
	30 years	42	27.1	106	68.4	7	4.5	
	≥40 years	33	28.4	80	69.0	3	2.6	
Education	No educational certificate	8	66.7	4	33.3	0	0.00	0.048
	Secondary or below	30	28.8	72	69.2	2	1.9	
	Bachelor's degree and postgraduate	77	25.2	217	71.1	11	3.6	
Occupation	Clerk	41	25.0	118	72.0	5	3.0	0.233
	Healthcare worker	8	30.8	15	57.7	3	11.5	
	Housewife	52	30.6	114	67.1	4	2.4	
	Student	14	23.0	46	75.4	1	1.6	
Source of information	Healthcare workers	69	18.4	293	78.1	13	3.5	<0.001
	Friends and relatives	39	22.7	126	73.3	7	4.1	0.153
	Internet	25	32.5	48	62.3	4	5.2	0.199
	Television	23	35.9	37	57.8	4	6.3	0.037
	Books	17	37.0	28	60.9	1	2.2	0.274

Table 4 presents the association between socio-demographic characteristics (age, education, and occupation) and behavioral practice levels. The distribution of practice levels varies across different age groups. However, no statistically significant association is observed between age and behavioral practice levels (P = 0.567). There was a significant association between education and practice levels (P <0.001). Mothers with no educational certificate predominantly exhibit "low" attitude levels, while those with higher education levels show a more balanced distribution across practice levels. Finally, we did not find a significant association between occupation and practice levels overall (P = 0.331). However, certain occupations, such as health care workers, show notable variations in attitude distribution. The source of information did not differ significantly across the behavior and practice of the studied mothers except for the HCWs p = 0.002.

**Table 4:** Association between socio-demographic characteristics and the level of behavioral practice A p-value<0.05 is considered statistically significant.

Studied variables (N =421)		Behavioral practice level						
		Low		Moderate		High		P
		No.	%	No.	%	No.	%	
Age	< 20 years	4	28.9	9	64.3	1	7.1	0.567
	20 years	26	19.1	76	55.9	34	25.0	
	30 years	32	20.6	86	55.5	37	23.9	
	≥ 40 years	17	14.7	66	56.9	33	28.4	
Education	No educational certificate	10	83.3	1	8.3	1	8.3	<0.001
	Secondary or below	22	21.2	62	59.6	20	19.2	
	Bachelor's degree and postgraduate	47	15.4	174	57.1	84	27.5	
Occupation	Clerk	22	13.4	96	58.5	46	28.05	0.331
	Healthcare worker	5	19.2	13	50.00	8	30.8	
	Housewife	37	21.8	95	55.9	38	22.4	
	Student	15	24.6	33	54.1	13	21.3	
Source of information	Healthcare workers	40	14.4	158	57.0	79	28.5	0.002
	Friends and relatives	25	14.5	104	60.5	43	25.0	0.159
	Internet	18	23.4	36	46.8	23	29.9	0.173
	Television	17	26.6	31	48.4	16	25.0	0.195
	Books	6	13.0	28	60.9	12	26.1	0.571

## Discussion

Diarrheal disease remains a significant global health issue, particularly among infants and children [11]. Clinical researchers play a vital role in advancing our understanding of epidemiology, etiology, and interventions for diarrhea. Collaborative efforts are needed to improve access to clean water, promote proper sanitation practices, encourage breastfeeding, and ensure widespread availability of ORT. By addressing the gap in knowledge and behavior toward diarrhea, we can further reduce childhood mortality rates and enhance global health outcomes.

Of the participants, a high proportion (27.3%) had low knowledge scores. Overall, the majority of participants (69.6%) fit into the moderate group with only 3% of participants meeting the criteria for having a good knowledge score. The bulk of individuals (56.3%) had moderate behavioral practice scores. A noteworthy but lesser proportion (24.9%) had high practice scores.

The knowledge and behavioral practices of mothers regarding childhood diarrhea are crucial in preventing and managing this common health issue among children. Several studies have highlighted the determinants and factors associated with childhood diarrhea and the role of maternal knowledge and practices in its prevention and management. A systematic review and meta-analysis in Ethiopia found that mothers' handwashing practices were associated with childhood diarrhea [34]. Additionally, maternal literacy influenced hygienic practices, child feeding, weaning, and sanitation practices, which were important factors for childhood diarrhea [35]. Furthermore, environmental and behavioral practices such as unavailability of toilet facilities, improper solid waste disposal, and unprotected drinking water were significant factors in childhood diarrhea [36].

Studies have also emphasized the relationship between mothers' knowledge and behavior in preventing childhood diarrhea [37]. In Ethiopia, inadequate handwashing practices and limited knowledge of mothers on diarrhea were identified as factors for increasing the odds of childhood diarrhea at the individual level [38]. Moreover, the change in knowledge of childhood diarrhea could be due to variance in mothers' education levels [25].

In this study, mothers generally exhibit awareness of common causes of childhood diarrhea, including viral and bacterial infections, contaminated water, and poor sanitation. Maternal knowledge extended to preventive measures such as proper handwashing, exclusive breastfeeding, ensuring a clean water supply, and understanding the importance of vaccinations, particularly for rotavirus, which is essential for preventing diarrheal episodes.

Our findings also came along with a nationwide survey in Tanzania which highlighted the association between water, sanitation, and hygiene (WASH)-related behaviors and knowledge of childhood diarrhea, emphasizing the value of parental involvement in lowering morbidity and mortality among children [39]. Similarly, a study in Ethiopia identified determinants of childhood diarrhea, emphasizing the policy implications and insights for strengthening health intervention programs [36]. Furthermore, seeking healthcare for childhood diarrhea was associated with factors such as maternal age, household monthly income, expenditure for healthcare, and mother's knowledge about danger signs [40].

Maternal education was significantly associated with knowledge. Participants who lack literacy exhibit a greater percentage of low knowledge (66.7%, P = 0.048). Furthermore, maternal education and practice levels were significantly correlated (P < 0.001). Low-educated individuals predominantly exhibit "low" behavioral practice levels, while those with higher education levels show a more balanced distribution across practice levels.

The relationship between maternal education and the knowledge and behavioral practices of mothers regarding childhood diarrhea has been a subject of interest in public health research. Maternal education has been identified as a significant factor influencing the knowledge and practices related to childhood diarrhea. Studies have shown that higher maternal education levels are associated with better knowledge and management practices. For instance, a study in Egypt found that highly educated mothers exhibited better knowledge and management practices in dealing with fever in preschool children [41]. Furthermore, the effect of training provided to mothers on their practices and self-efficacy in preventing childhood diarrhea has been investigated. This study found that mothers who received education acquired cognitive behaviors at a sufficient level, indicating the positive impact of education on maternal practices and self-efficacy in preventing childhood diarrhea [42]. Additionally, maternal education was significantly associated with acute diarrhea among children aged 2-5 years in Pakistan [43].

Overall, our results match findings from previous literature which suggested a strong relationship between maternal education and the knowledge and behavioral practices of mothers regarding childhood diarrhea. Higher maternal education levels are associated with better knowledge, management practices, and self-efficacy in preventing childhood diarrhea. These findings underscore the importance of educational interventions and the involvement of mothers in promoting effective preventive measures and management strategies for childhood diarrhea.

From our study, we found that sources of information from HCWs and TV had significant differences across the knowledge of the studied mothers. While information from HCWs differs significantly across mothers’ behavior. Mothers often exhibit prompt health-seeking behavior upon recognizing diarrhea symptoms. However, barriers such as financial constraints and limited access to healthcare facilities may impede timely treatment, highlighting the need for targeted interventions. Furthermore, disparities exist in the awareness and uptake of vaccines targeting diarrhea. Factors such as maternal education and socioeconomic status influence the likelihood of vaccination, underscoring the importance of targeted immunization campaigns.

Interventions and recommendations

Educational initiatives tailored to mothers have demonstrated positive impacts on knowledge enhancement and the adoption of recommended practices. Culturally sensitive approaches are crucial for the success of these programs. Also, improving access to healthcare services, particularly in rural areas, is vital for ensuring timely treatment and prevention of childhood diarrhea. Integrating healthcare into community settings can enhance accessibility.

Strength and limitations

The first limitation of the study was that we did not use a probability sampling design, which may hinder the generalization of the study findings. Second, we collected data online through the circulation of the questionnaire on social media, thus illiterate mothers who cannot read were not able to participate. However, we employed a large sample and addressed vast and representative public sectors during the sampling procedure. To the best of our knowledge, this is the first study to address maternal knowledge and practice toward childhood diarrhea in the Northern region of Saudi Arabia. However, further research is needed to examine the long-term impact of maternal knowledge and practices on the overall health and well-being of children beyond the immediate episode of diarrhea are needed to be conducted.

## Conclusions

Improving the knowledge and behavioral practices of mothers regarding childhood diarrhea is crucial for reducing the burden of this preventable disease. The findings highlight the importance of targeted education programs and community-based interventions to improve mothers' knowledge and promote appropriate behavioral practices related to childhood diarrhea that ultimately will lead to improved health outcomes for children globally. Tailoring interventions to the specific social and cultural contexts of communities is essential for effective implementation. Acknowledging and addressing cultural beliefs and practices can enhance the acceptance of health recommendations.

## Appendices

Data collection tools

I. Socio-demographic characteristics

1- Age of mother (In years)

2- Do you live in Arar?

·       Yes

·       No

3- Education

·       Illiterate

·       School education or less

·       Diploma/bachelor’s degree

·       postgraduate

4- Occupation

·       Housewife

.       Employer 

·       Health staff

·       Student

·       Other

5- Do you have a child or more under 5 years old?

·       Yes

·       No

6- Gender of the child

·       Male

·       Female

7- Age of the child (In years: months)

II. Knowledge about the definition, causes, and mode of transmission of diarrhea

1- Diarrhea is defined as passing more liquid or loose stools each day or more frequently than is typical for the individual.

Yes

No

2- The correct causative agent (virus, bacteria or protozoa)?

Yes

No

3- Diarrhea can be transmitted by house flies.

Yes

No

4- Diarrhea be transmitted by unwashed vegetables and fruits?

Yes

No

5- Sensitivity to some foods and medicines can cause diarrhea.

Yes

No

6- The use of antibiotics for a long duration can cause diarrhea.

Yes

No

7- Contaminated shells of eggs can cause diarrhea.

Yes

No

8- Teething in infants can cause diarrhea.

Yes

No

9- Maintenance of Breastfeeding can lead to diarrhea.

Yes

No

10- Falling from a height can cause diarrhea.

Yes

No

11- Evil eye can cause diarrhea?

Yes

No

12- Eating hot food can cause diarrhea.

Yes

No

13- Weather changes can cause diarrhea?

Yes

No

III. General knowledge about a clinical picture of diarrhea

1- Which of the following is a symptom of diarrhea?

(You can choose more than one)

·       Sunken eyes

·       Dryness and shrinkage of skin

·       Thirst

·       Dry hair

·       Decreased salivation.

·       Decrease urine.

·       Drowsiness or decreased consciousness

IV. Knowledge about prevention of diarrhea

1- Diarrhea is a preventable disease?

Yes

No

2- Personal hygiene and hand washing prevent diarrhea.

Yes

No

3- Some viral and bacterial vaccines prevent diarrhea.

Yes

No

4- Does maintenance of Breastfeeding protect against diarrhea?

Yes

No

5- Avoiding dairy products can prevent diarrhea?

Yes

No

6- Does using boiled or sterile water prevent diarrhea?

Yes

No

V. Knowledge about the treatment of diarrhea

1- Treatment of diarrhea availability?

Yes

No

2- Diarrhea should be treated with antibiotics.

Yes

No

3- Oral rehydration solutions (ORS) is important in treatment?

Yes

No

4- Oral rehydration solutions (ORS) should be given once per day?

Yes

No

5- Oral rehydration solutions (ORS) should be given several times a day?

Yes

No

6- Oral rehydration solutions (ORS) can be dissolved in juice?

Yes

No

7- Oral rehydration solutions (ORS) should be dissolved in clean sterile water.

Yes

No

8- Diarrhea can be treated only by herbal preparations?

Yes

No

9- Diarrhea can be treated only by bed rest?

Yes

No

10- Does solid food (potatoes, apples, carrots) help in the treatment of diarrhea?

Yes

No

11- Breastfeeding should be stopped during diarrhea?

Yes

No

12- Constipating drugs can be used during the treatment of diarrhea?

Yes

No

VI. Behavioral practice of diarrhea

1- Practice using antibiotics during the treatment of diarrhea

Yes

No

2- Practice using oral rehydration solutions (ORS) during the treatment of diarrhea

Yes

No

3- The practice of giving oral rehydration solutions (ORS) only once per day

Yes

No

4- Practice of giving oral rehydration solutions (ORS) several times a day

Yes

No

5- Practice of dissolving oral rehydration solutions (ORS) in juice

Yes

No

6- Practice dissolving oral rehydration solutions (ORS) in clean sterile water

Yes

No

7- Practice of treatment of diarrhea only with herbal preparations

Yes

No

8- Practice of treatment of diarrhea only by bed rest

Yes

No

9- Practice of treatment of diarrhea in hospital

Yes

No

10- Practice of treatment of diarrhea at home

Yes

No

11- Practice Solid food in the treatment of diarrhea

Yes

No

12- Practice stopping breastfeeding during diarrhea

Yes

No

13- Practice the of use Constipating drugs during the treatment of diarrhea

Yes

No

14- The practice of Washing of hands

Yes

No

15- Practice of washing of raw vegetable and fruits

Yes

No

VII. Source of information about diarrhea

Which sources do you prefer to obtain information about childhood diarrhea? (You can choose more than one source)

·       Television

·       Newspaper

·       University or school

·       Internet

·       Health worker

·       Friends and relatives

·       Books
